# Allergy protection at farms—just a myth?

**DOI:** 10.1002/iid3.378

**Published:** 2020-11-17

**Authors:** Matthias Wjst

**Affiliations:** ^1^ Institute of Lung Biology and Disease, Helmholtz Zentrum Muenchen, German Research Center for Environmental Health (GmbH) München Germany; ^2^ Institut für Medizinische Informatik, Statistik und Epidemiologie Technische Universität München München Germany

Allergy on farms has a long history. “Hayfever,” one of the main allergic outcomes, has an origin in the farming community while also asthma is increased in farmers due to exposure to grain dust, animal dander and various chemicals,^1^, ^2^ Nevertheless Blackley^3^ noted already in 1873 that “*these statistics of the occupations of hay‐fever patients bring out prominently the very curious circumstance that the persons who are most subjected to the action of pollen belong to a class which furnished the fewest cases of the disorder, namely, the farming class*”. This paradox has been rediscovered in 1989 by Gassner in the Canton of St. Gallen in the Swiss Alps,^4^ followed by studies of Braun‐Fahrländer^5^ among others. 30 years of research at Alpine farms within the framework of the “hygiene hypothesis,”^6^, ^7^ is raising now the question if allergy protection at farms is just a modern myth.

The main objection against the farming hypothesis is the interpretation of a negative statistical association as a “protective” effect. Only after thorough exclusion of alternative explanations, this interpretation may be justified. Unfortunately previous farming studies did not exclude other reasons but always followed the same line of arguments that (i) allergy and asthma prevalence is lower in the farming environment, (ii) that this particular environment has different exposure conditions, and (iii) these different exposure conditions are responsible for the lower allergy prevalence. (i) and (ii) are certainly true, but (iii) may be false if both statements are not correlated at all, for example, if the propositional calculus is being wrong.

A world‐wide study at least showed that farming is a risk but not a protective factor.^8^ In this study of 44 centers from 61 countries with 388,811 6‐ to 7‐year‐old children, an odds ratio of 1.2 was shown for hayfever when exposed to farm animals. Also more detailed studies could not replicate the lower allergy prevalence.^9^ From the list of nearly two dozen published “causal” factors (listeria, ascaris, toxoplasma, rubella, eurotium, penicillium, acinetobacter, corynebacterium, endotoxin, spores, horse, dog, pig, cow, milk, whey, silage, dung hills, and cleanliness), none could be verified in the general population. Even the most recent claim of a protective farm index^10^ looks more like “cherry picking” in a null result as no bacterial species could be identified. Farming studies have never been pre‐registered nor are any datasets available for independent review.

Could therefore be a rather trivial reason for the lower allergy prevalence in farms? Most recently not only confounding but also colliding has received an increased interest. While confounding describes the action of a third variable on exposure and outcome, colliding describes the selection bias that stems from conditioning on a variable that is itself influenced by exposure and outcome,^11^, ^12^ Colliding may be expected in studies at rural areas where selective migration over generations may have introduced a healthy worker bias with less allergy genes in today's farming population.^13^ Already one of the first papers already showed that not only children but also less than half of their parents had a history of allergic rhinitis.^5^ The literature on a healthy worker bias in rural areas is not fully clear while the largest study concluded^14^ that “*selective migration over generations could therefore have contributed to a healthy worker effect with less atopy and less severity of symptoms in the farming population today*.” Comparing farm children with their neighborhood may therefore introduce a spurious association (Figure 1). In addition, also cryptic confounding is likely as it is known that farm children receive less vitamin D avoiding a known risk factor for allergy.^15^

**Figure 1:**
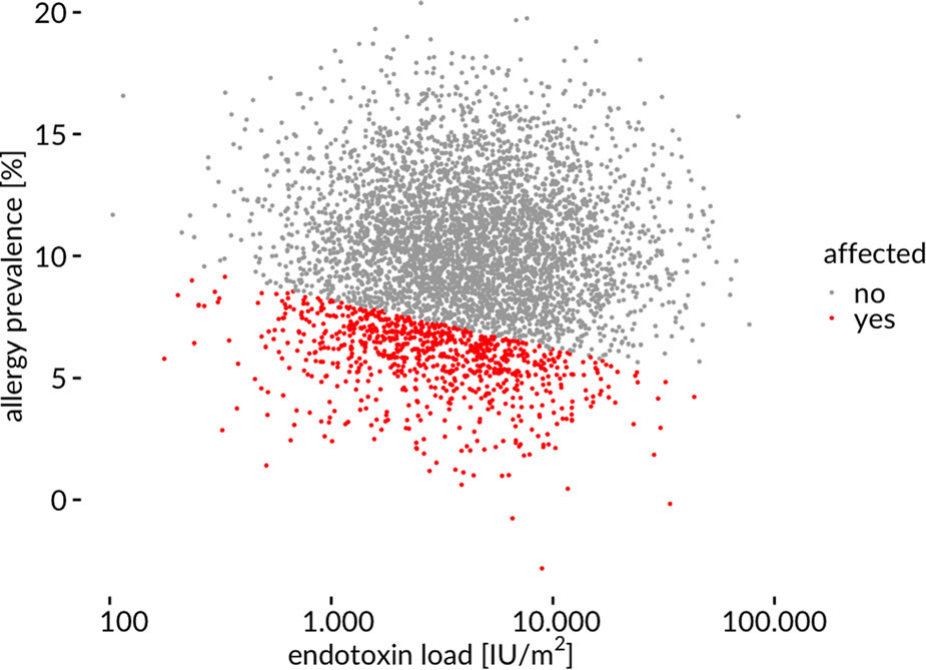
The figure shows how sampling bias at rural sites may have further amplified a spurious association. This is a hypothetical example of two normally distributed variables, allergy prevalence and endotoxin load, that are not associated with each other. Farming studies have initially been started due to the observation that there is less allergy at rural sites. Sampling at farms selects children with high endotoxin exposure and rather low allergy prevalence as they originate from healthy parents. As reference sample usually nonfarm children from rural neighborhoods are selected, including more individuals with a lower endotoxin exposure but also higher disease prevalence, introducing therefore a negative association

Although a true effect is questionable from an epidemiological viewpoint, there are some animal studies that are seemingly supporting the farm effect. It turns out, however, that we are amidst a replication crisis where inbred mouse strains are having their own problems,^16^, ^17^ in particular when it comes to immunological readouts.^18^ This may be particular true if mice are kept indoors under allergen deprivation and are fed on a vitamin D diet. Results in mice therefore may be as questionable^19^ as the epidemiological results. Cow stables have a long tradition in generating myths.^20^ “Allergy protection” on farms in its present form looks like another myth at least until convincing arguments can be provided that this is not just an epidemiological fallacy.

## References

[1] Rask‐Andersen A. Asthma increase among farmers: a 12‐year follow‐up. Ups J Med Sci. 2011;116:60‐71. 20812893 10.3109/03009734.2010.503287 PMC3039762

[2] Sigsgaard T , Basinas I , Doekes G , et al. Respiratory diseases and allergy in farmers working with livestock: an EAACI position paper. Clin Transl Allergy. 2020;10:29. 32642058 10.1186/s13601-020-00334-x PMC7336421

[3] Blackley CH . Experimental researches on the causes and nature of catarrhus aestivus (hay‐fever or hay‐asthma). Baillière, Tindall & Cox, 1873. https://books.google.de/books/about/Experimental_Researches_on_the_Causes_an.html

[4] Gassner M. Allergie und Umwelt. Allergologie. 1989;12:492‐502.

[5] Braun‐Fahrländer C , Gassner M , Grize L , et al. Prevalence of hay fever and allergic sensitization in farmer's children and their peers living in the same rural community. SCARPOL team. Swiss study on childhood allergy and respiratory symptoms with respect to air pollution. Clin Exp Allergy. 1999;29:28‐34. 10051699 10.1046/j.1365-2222.1999.00479.x

[6] von Mutius E , Vercelli D. Farm living: effects on childhood asthma and allergy. Nat Rev Immunol. 2010;10:861‐868. 21060319 10.1038/nri2871

[7] Ege M , Rompa S. The hygiene hypothesis of allergy and asthma. Encycl Immunobiol. 2016;5:328‐335.

[8] Brunekreef B , von Mutius E , Wong GK , Odhiambo JA , Clayton TO , ISAAC Phase Three Study Group . Early life exposure to farm animals and symptoms of asthma, rhinoconjunctivitis and eczema: an ISAAC phase three study. Int J Epidemiol. 2012;41:1‐9. 22287135 10.1093/ije/dyr216

[9] Ege MJ , Frei R , Bieli C , et al. Not all farming environments protect against the development of asthma and wheeze in children. J Allergy Clin Immunol. 2007;119:1140‐1147. 17349684 10.1016/j.jaci.2007.01.037

[10] Kirjavainen PV , Karvonen AM , Adams RI , et al. Farm‐like indoor microbiota in non‐farm homes protects children from asthma development. Nature Med. 2019;25:1089‐1095. 31209334 10.1038/s41591-019-0469-4 PMC7617062

[11] Munafò MR , Tilling K , Taylor AE , Evans DM , Davey , Smith G. Collider scope: When selection bias can substantially influence observed associations. Int J Epidemiol. 2018;47:226‐235. 29040562 10.1093/ije/dyx206 PMC5837306

[12] Rohrer JM . Thinking clearly about correlations and causation: Graphical causal models for observational data. Adv Methods Pract Psychol Sci. 2018;1:27‐42.

[13] Kim KW , Ober C. Lessons learned from GWAS of asthma. Allergy Asthma Immunol Res. 2019;11:170‐187. 30661310 10.4168/aair.2019.11.2.170 PMC6340805

[14] Bråbäck L , Hjern A , Rasmussen F. Selective migration contributes to a healthy worker effect in the farming population. J Clin Epidemiol. 2006;59:102‐103. 16360568 10.1016/j.jclinepi.2005.08.003

[15] Hyppönen E , Sovio U , Wjst M , et al. Infant vitamin d supplementation and allergic conditions in adulthood: northern Finland birth cohort 1966. Ann NY Acad Sci. 2004;1037:84‐95. 15699498 10.1196/annals.1337.013

[16] Collins FS , Tabak LA . Policy: NIH plans to enhance reproducibility. Nature. 2014;505:612‐613. 24482835 10.1038/505612a PMC4058759

[17] Voelkl B , Altman NS , Forsman A , et al. Reproducibility of animal research in light of biological variation. Nat Rev Neurosci. 2020;21:384‐393. 32488205 10.1038/s41583-020-0313-3

[18] Wenzel S , Holgate ST . The mouse trap: it still yields few answers in asthma. Am J Respir Crit Care Med. 2006;174:1173‐1176. 17110654 10.1164/rccm.2609002

[19] Garner JP . The significance of meaning: why do over 90% of behavioral neuroscience results fail to translate to humans, and what can we do to fix it. ILAR J. 2014;55:438‐456. 25541546 10.1093/ilar/ilu047 PMC4342719

[20] Boylston AW . The myth of the milkmaid. N Engl J Med. 2018;378:414‐415. 29385360 10.1056/NEJMp1715349

